# Radioanatomical evaluation of the subtympanic sinus in children under five years old and its clinical implications - high resolution computed tomography study

**DOI:** 10.1007/s00276-024-03508-5

**Published:** 2024-10-23

**Authors:** Tomasz Wojciechowski, Stanisław Szeliga, Tymon Skadorwa

**Affiliations:** 1 Department of Otorhinolaryngology, Head and Neck Surgery, Medical University of Warsaw, 1a Banacha St, Warsaw, 02097 Poland; 2 Department of Descriptive and Clinical Anatomy, Medical University of Warsaw, 5 Chałubińskiego St, Warsaw, 02004 Poland; 3 Department of Pediatric Neurosurgery, Bogdanowicz Memorial Hospital for Children, 4/24 Niekłańska St, Warsaw, 03924 Poland; 4 Department of Pediatric Neurosurgery, Medical University of Warsaw, 63A Żwirki i Wigury St, Warsaw, 02091 Poland

**Keywords:** Subtympanic sinus, Retrotympanum, Middle ear, Temporal bone, Endoscopic ear surgery, Temporal bone computed tomography

## Abstract

**Purpose:**

This study aimed to evaluate subtympanic sinus (STS) and its vicinity in high-resolution computed tomography (HRCT) scans of children under five years old with non-diseased temporal bones.

**Material and method:**

We divided the whole group into children under 24 months of age (first stage of pneumatisation development) and between 25 and 60 (second stage). We have determined the width of the entrance to STS, depth of the STS, type in relation to facial nerve according to Anschuetz classification, the pneumatisation of posterior and medial air cell tracts, and jugular bulb position. All the HRCTs (280 temporal bones) were analyzed according to the multiplanar reconstruction protocol with symmetrization.

**Results:**

STS’s mean width and depth were 2.71 ± 0.60 mm and 3.26 ± 1.11 mm, respectively. The most common STS type was type A (59.3%), followed by type B (30.7%) and type C (10%). The posterior air cell tract (retrofacial cells) was present in 39.3%. The medial air cell tract (hypotympanic cells) was present in 30.7% The jugular bulb position affected the final shape of STS in 17.5%.

**Conclusion:**

The results support the necessity of the classification for the STS. Our study may help with surgical planning regarding endoscopic ear procedures and gives a broader understanding of how pneumatization or jugular bulb might correlate with the final shape of the retrotympanum. The historical remarks track the term’s origin for clarity in research and respect for earlier investigators.

## Introduction

**Fig. 1:**
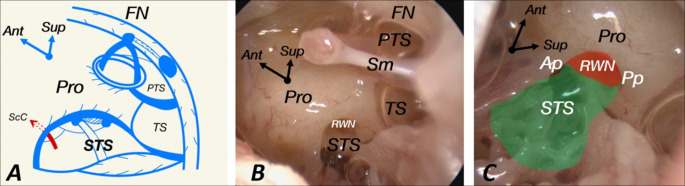
**A** Scheme of medial recesses of retrotympanum **B** Left endoscopic view of medial recesses of retrotympanum with use of 0-degree scope **C** Detailed left endoscopic view of inferior retrotympanum with use of 0-degree scope slightly clockwise rotated in comparison to **image B**. Ant – anteriorly, Sup – superiorly, FN - facial nerve, Pro – promontory of cochlea, PTS – posterior tympanic sinus, TS – tympanic sinus, STS – subtympanic sinus, ScC – subcochlear canaliculus, Sm – tendon of stapedius muscle, RWN – round window niche, Ap – anterior pillar, Pp – posterior pillar; photos gathered from the Department’s collection.

The shape of the posterior wall of the tympanic cavity is very complex, and several bony projections like ridges, bridges, crests, and eminences may be found. These structures divide the retrotympanum – the whole space located posteriorly to the fibrocartilaginous ring – into smaller spaces, recesses, and sinuses. The mastoid part of the facial nerve divides the retrotympanum into lateral and medial spaces. The lateral spaces are the facial sinus and lateral tympanic sinus, and together, they form the so-called facial recess. The subiculum promontorii separate medial spaces into superior and inferior retrotympanum. Within the superior retrotympanum sinus tympani and the posterior tympanic sinus are found. The inferior retrotympanum is a transitional area located between the sinus tympani and the hypotympanum. The limits are contractual as the posterior wall merges with the inferior, forming a rather blunt or even round floor (Fig. 1.).

**Fig. 2:**
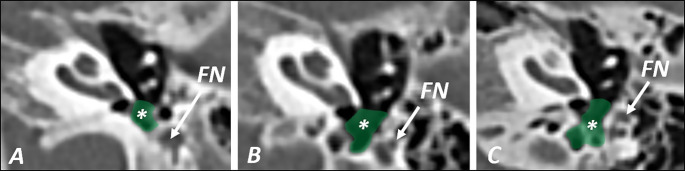
Different types of the subtympanic sinus; **STS type A** is very shallow and does not reach the anterior border of the facial nerve in transverse sections, **type B** reaches the circumference of the facial nerve but does not extend beyond its posterior border and **type C**, defined as very deep, extends beyond the posterior circumference of the facial nerve. All the scans are made in tilted axial plane and show left temporal bones

The subtympanic sinus (STS) is a bony area within the inferior retrotympanum, antero-inferiorly to the tympanic sinus, medial to the styloid prominence, and posterior to the cochlear promontory. The pyramidal eminence is lateral to the subtympanic sinus, separating it from the facial recess [16]. The sinus is divided from the tympanic sinus by the subiculum promontorii, usually appearing as a bony ridge that extends from the posterior pillar of the round window niche to the styloid prominence. The finiculus forms an anteroinferior limit of the subtympanic sinus, a bony ridge rising from the styloid prominence and extending to the anterior pillar of the round window niche [16]. The fustis is a bony structure traverses the sinus while it arises from the styloid prominence in the posterior aspect of the sinus and directs medially towards the round window niche [18]. Anschuetz et al. [3] suggested a novel surgical and radiological classification of the subtympanic sinus, regarding the extension of the fundus of the sinus according to the mastoid portion of the facial nerve. Three types (A-C) of the subtympanic sinus are defined. Type A refers to the sinus having an extension corresponding with the medial border of the facial nerve. In type B, the sinus extends to the medial limit of the facial nerve but without posterior extension. Type C subtympanic sinus expands posterior to the facial nerve, reaching the mastoid cavity (Fig. 2.) [3].

The jugular bulb is the first part of the internal jugular vein and is placed in the jugular fossa. The jugular bulb develops after the birth until the second year of life. The asymmetry of jugular bulb with its right side domination is often reported as the more common configuration than symmetrical jugular bulbs. High-riding jugular bulb may also be adjacent to the posterior semicircular canal or hypotympanum. In some situations, the air cell tracts may separate the jugular bulb from neighboring structures. Marchioni et al. [16] suggest the shape of STS may be affected by presence of high jugular bulb and the depth of the STS may depend on pneumatisation of retrotympanic spaces and cell tract around these.

Cholesteatoma is a form of chronic otitis media characterized by the presence of squamous cells “in the wrong place”, i.e., inside the tympanic cavity. The disease is particularly aggressive in children. The place of origin may vary – cholesteatoma may develop from the retraction pocket of pars flaccida of the tympanic membrane or marginal perforation of pars tensa. Depending on these factors, the routes of spread may be different. Cholesteatoma from pars flaccida invades the epitympanum with the ossicles, the facial sinus, and aditus ad antrum. Pars tensa cholesteatoma may invade the mesotympanum, facial recess, sinus tympani, and eventually subtympanic sinus and hypotympanum. The subtympanic sinus is a gateway to the round window niche located anteromedial to the sinus, the subcochlear canaliculus, and semicellulae of hypotympanum. During cochlear implantation, it is essential to localize all the landmarks of subtympanic sinus and to know its topography to avoid placement of the electrodes’ chain to air spaces rather than to the round window.

In light of the clinical context and the lack of data in the literature, we set up an investigation of subtympanic sinus in computed tomography scans as it is a routine examination before cochlear implantation and cholesteatoma surgery in children. Our study aimed to evaluate the type of the subtympanic sinus according to Anschuetz classification (1), to measure the width and depth of the subtympanic sinus (2), assess its relationship with pneumatization routes around the sinus (3) and whether the final shape of the subtympanic sinus is affected by the jugular bulb position (4). The additional goal was to track the historical information about the recess we now call the subtympanic sinus (5).

## Material and methods

This was a retrospective study for which we have selected an anonymized set of High-Resolution Computed Tomography (HRCT) scans from the Department of Paediatric Neurosurgery, Bogdanowicz Memorial Hospital for Children. All the scans came from the period between February 2016 and June 2018. We obtained the scans due to clinical indications (neurosurgical - post-traumatic) using a CT scanner Siemens Somatom Emotion (Siemens Healthcare, Erlangen, Germany) with the following settings: slice thickness 0,5 mm; the exposition was performed with various source voltage (from 90 to 120 kV) and current (from 180 to 270 mA) depending on the age of the patient. The whole population involved in the analysis was of Caucasian race (the ethnic group of native Poles). The age of patients included in the study ranged from 4 to 60 months (5 years old). We have excluded from the study all the images containing any temporal bone pathology (bony labyrinth developmental pathologies, fractures, fluid in the middle ear spaces). Overall, the analyzed group consisted of 140 sets of images (68 females, 72 males, 280 temporal bones). We have divided them into two smaller groups: children from 4 to 24 months of age (196 temporal bones – Group I) and from 25 to 60 months of age (84 temporal bones – Group II). All scans were analyzed with the software RadiAnt DICOM Viewer 2022.1 (64-bit; Medixant, Poznan, Poland). The window level was set to 500 Hounsfield Units (HU), and the window width was 3500 HU. All the measurements were performed together by two scientists (TW and SS), and in case of any differences, the consensus was made after discussion.

**Fig. 3:**
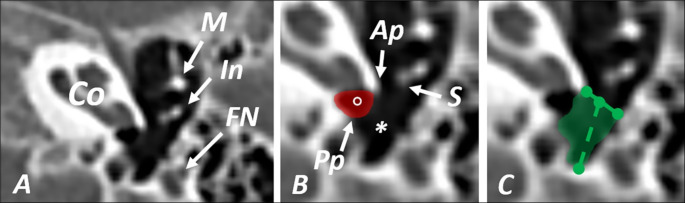
**a** General view of the area of measurements; Co – cochlea, M – manubrium of malleus, In – long crus of incus, FN – facial nerve **b** Detailed view of the area of subtympanic sinus; Ap – anterior pillar, S – head of stapes, Pp – posterior pillar, asterisk – subtympanic sinus, red area with circle – round window niche **c** The green area - subtympanic sinus, green line – width of the entrance to the subtympanic sinus, green dotted line – depth of subtympanic sinus; All the scans are made in tilted axial plane and show left temporal bones

As the retrotympanum is a highly complex area, we have precisely followed the protocol of image analysis with the multiplanar reconstruction tool. At first, we set the tilted horizontal plane in order to visualize the signet ring appearance of lateral semicircular bilaterally. At the round window niche level, we have been assessing the type of subtympanic sinus according to the classification proposed by Anschuetz et al. [3]. In order to accurately evaluate the type of subtympanic sinus, one must check three to five scans superiorly and inferiorly. This procedure prevents the mistake of assessing the tympanic sinus instead and allows the following of the mastoid part of the facial nerve. Then, we took the measurements at the level of the round window niche when the anterior and posterior pillars were visualized. We have defined the width of the entrance to the subtympanic sinus as the measurement from the anterior pillar to the pyramidal crest, styloid eminence, or most prominent bony structure limiting the access to the STS. The depth of the STS was measured from the middle point of the latter line to the deepest posterior part of the STS on the image (Fig. 3).

**Fig. 4:**
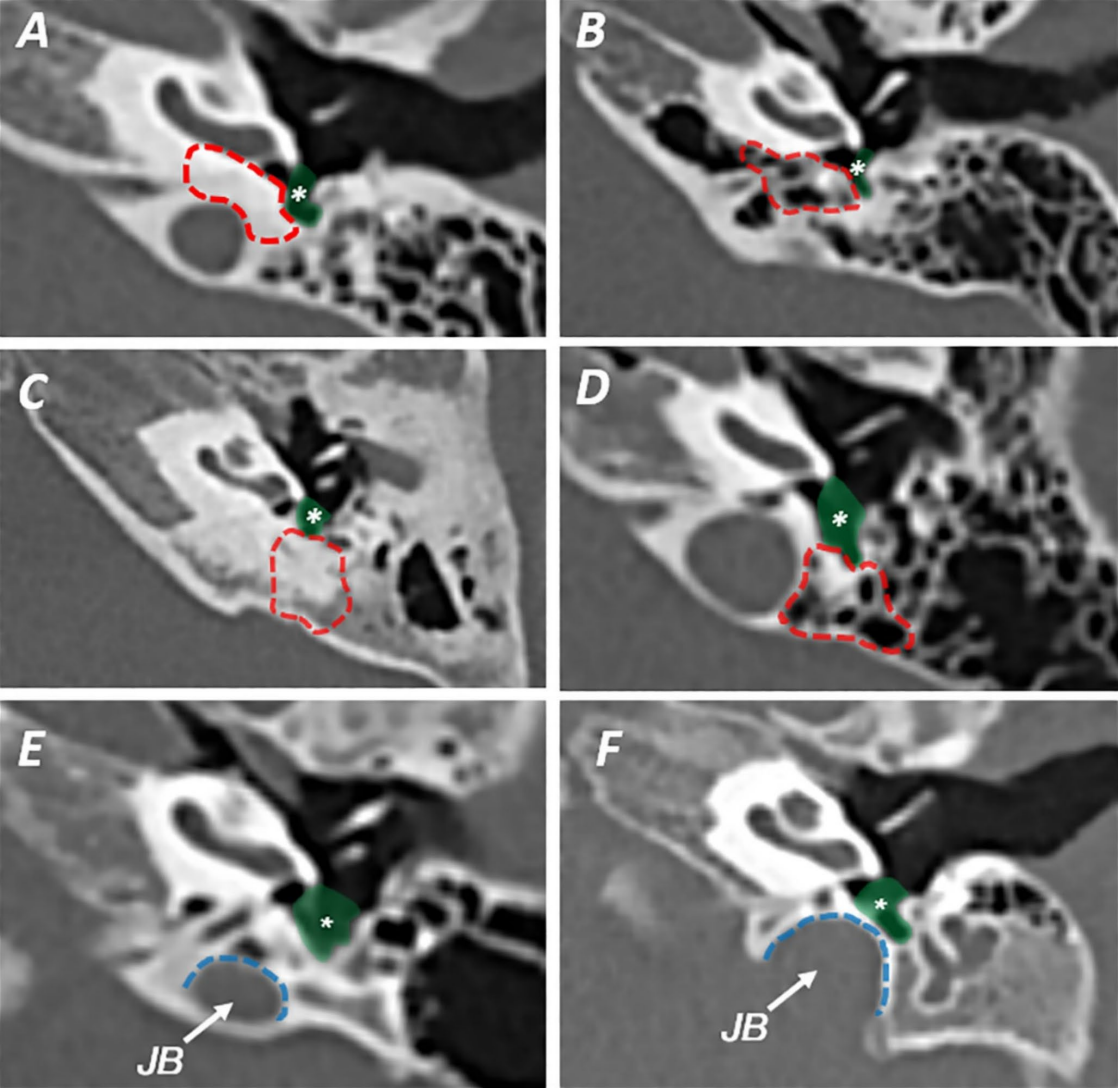
**A** air cells absent medially to the STS **B** air cells present medially to the STS **C** air cells absent posteriorly to the STS **D** air cells absent posteriorly to the STS **E** jugular bulb (JB) distant from the bottom of STS **F** jugular bulb narrowing the STS; asterisk and green area – subtympanic sinus; All the scans are made in tilted axial plane and show left temporal bones

In the next step, we investigated images according to Allam’s classification for the presence of air cell tracts posteriorly (retrofacial cells) and medially (hypotympanic cells) to the STS. The absence of pneumatization was defined as no air cells in a 5 mm radius from STS. Finally, we have been checking for jugular fossa presence and position and whether it influences the shape of the STS (Fig. 4.).

The mean value, standard deviation (SD), min-max range, and normality of the distribution with the Shapiro-Wilk test were calculated for each measured parameter. We have compared all parameters between sides and gender. We have tested the observed differences with Student’s t-test for parametric and Mann-Whitney U-test for nonparametric variables. A chi-squared test was used to assess the differences between the qualitative variables. Relationships between quantitative and qualitative variables were assessed with Spearman’s rho correlation coefficient and Cramér’s V. The *p* < 0.05 was considered significant for all comparisons. The statistical analysis was performed with TIBCO DataScience/Statistica software by StatSoft Europe, version 13.3 PL for Microsoft Windows 10 Pro.

The study has been approved by the Ethics Committee of Medical University of Warsaw (decision number AKBE/187/2019), and abides by the 1964 Helsinki Declaration and its later amendments or comparable ethical standards. We have followed the AQUA checklist to ensure the clarity of this anatomical report.

## Results

Subtympanic sinus was found in all assessed scans. From 280 analyzed temporal bones the most common type was type A (59.3%), followed by type B (30.7%) and type C (10%). There were statistically significant differences between distribution of each type of the STS (chi-squared test, *p* < 0.0001). Type A and B was observed more frequently on the right side (chi-squared test, not significant). Both boys and girls were dominated by STS type A, followed by type B and type C (chi-squared test, not significant). The differences in numbers of particular types of ST among age groups were significant (ch-squared test, *p* = 0.0115). Detailed distribution according to side and gender is presented in the Table 1.

**Table 1:** The number of identified types of STS according to side, gender and age

		Type A	Type B	Type C	Total
side	left right	82 (58.6%) 84 (60%)	41 (29.3%) 45 (32.1%)	17 (12.1%) 11 (7.9%)	140 (50%) 140 (50%)
gender	females males	83 (61%) 83 (57.6%)	42 (30.9%) 44 (30.6%)	11 (8.1%) 17 (11.8%)	136 (48.6%) 144 (51.4%)
age	4–24 m 25–60 m	105 (53.6%) 61 (72.6%)	68 (34.7%) 18 (21.4%)	23 (11.7%) 5 (6%)	196 (70%) 84 (30%)
Total		166 (59.3%)	86 (30.7%)	28 (10%)	280 (100%)

The average width of the entrance to STS for the whole studied group was 2.71 ± 0.60 mm (1.19–4.86 mm) and the average depth of STS was 3.26 ± 1.11 mm (1.10–8.43 mm). We have found the width of STS varied significantly between boys and girls (*p* = 0.0193) but not the depth (*p* = 0.0814). We have not found the statistically significant differences in width and depth of STS between sides. Mean values, SD and min-max ranges for all measured parameters with respect to types of STS, gender, side and age groups are presented in Table 2. We have found no statistically significant differences in values of the STS width and depth between age groups.

**Table 2:** Mean values, SD and min-max range of the measured parameters according to the type of STS

		Width mean ± SD (range)	Depth mean ± SD (range)
side	Left	2.69 ± 0.60 (1.21–4.21)	3.21 ± 1.10 (1.10–6.99)
	Right	2.73 ± 0.61 (1.19–4.86)	3.31 ± 1.12 (1.23–8.43)
gender	Females	2.62 ± 0.58 (1.21–4.28)	3.13 ± 1.06 (1.10–6.27)
	Males	2.79 ± 0.62 (1.19–4.86)	3.38 ± 1.16 (1.23–8.43)
age	4–24 m	2.69 ± 0.59 (1.19–4.86)	3.32 ± 1.09 (1.23–8.43)
	25–60 m	2.74 ± 0.65 (1.30–4.28)	3.11 ± 1.16 (1.10–6.99)
type	A	2.69 ± 0.60 (1.21–4.28)	2.69 ± 0.73 (1.10–4.79)
	B	2.71 ± 0.56 (1.49–4.13)	3.76 ± 0.80 (1.95–6.27)
	C	2.78 ± 0.78 (1.19–4.86)	5.06 ± 1.12 (3.31–8.43)
total		2.71 ± 0.60 (1.19–4.86)	3.26 ± 1.11 (1.10–8.43)

We also have found the statistically significant deviations (chi-squared test, *p* < 0.0001) in depth of STS between all the three types across the whole studied population (Cramér’s V = 0.52, *p* = 0.0361).

From the whole group of bones (*n* = 280) the posterior air cell tract (retrofacial cells) was present in 39,3% and it has increased from 34.2% in group below 24 month of age (Group I) to 51.2% in age group II (25–60 months old). The difference between age group was statistically significant (*p* = 0.0076). The medial air cell tract (hypotympanic cells) was present in 30.7% and it has increased from 29.1% in age group I to 34.5% in age group II. The difference between age group was not statistically significant (*p* = 0.3657). The presence of the posterior and medial air cells tracts was not correlated with the depth of STS (chi-squared test, *p* = 0.2093 and *p* = 0.3386 accordingly). The jugular bulb position affected the final shape of STS in 15.3% in age group I and 22.6% in age group II (17.5% in whole group) but the difference was not statistically significant (chi-squared test, *p* = 0.1399).

## Discussion

### Historical remarks

Prof. Gary Wise once wrote “(…) we must recognize that history does not exist as a separate entity to be studied in a vacuum. Instead, it is a vital component of science, a component which can guide us toward the ever-shifting definition of truth.” [29]. We have decided to track the history of the terminology regarding tympanic cavity spaces between the facial nerve and the cochlea. This is because the unification of terminology is paramount for clear communication in anatomy and surgery.

The term “sinus subtympanicus” was first used in 2010 by Daniele Marchioni and his team from Modena [16]. In their paper, they have revised the anatomy retrotympanum for purposes of endoscopic ear surgery. Sinus subtympanicus replaced the term “area concamerata,” not that widely used in the pre-endoscopic era of ear surgery, and it now serves as an antechamber to round window niche and subcochlear canaliculus. The whole area between the subiculum and finiculus in endoscopic ear surgery is called the inferior retrotympanum. It is the transitional space between the posterior and inferior walls of the tympanic cavity.

Until the time of increased popularity of endoscopic ear surgery and better means of intraoperative visualization, the aural surgeons used the scheme of retrotympanum proposed by Bruce Proctor in 1969 [21]. Although he never used the term retrotympanum, he divided posterior tympanum into lateral and medial spaces and brought attention to the variability of bony crests projecting from styloid eminence/complex. He claimed the lateral spaces to the pyramidal eminence are the facial sinus and lateral tympanic sinus. He also described the ponticulus projecting from pyramidal eminence toward the cochlear promontory, dividing the medial spaces into smaller recess - posterior tympanic sinus and greater - sinus tympani. Proctor continued his work and published another paper on the round window niche with his colleagues in 1986 [22]. He then recognized the bony projections delineating a space between sinus tympani and hypotympanum, which he named “area concamerata”. According to him, the area concamerata was located between subiculum promontorii and sustentaculum promontorii (the latter was renamed to finiculus by Marchioni and his team, 2010) [16]. Area concamerata was divided by a bony crest leading to the round window niche Proctor named fustis. Its variations was also investigated by Marchioni and his team [18]. On the two sides of the fustis, recesses of different depths were located, and Proctor called them sinus concamerata medialis et lateralis. The first one was adjacent to postis posterior and served as the fundus of round window niche. The second one was not constant and difficult to divide from the hypotympanum. These days we know finiculus may be absent and then there is no clearly visible bony crest located anteriorly to the fustis serving as border between anterior and inferior walls of tympanic cavity. Proctor was not the only one who was investigating this area that time – Savic and Djeric published their work on sinus hypotympanicus in 1987 [23]. As far as one may compare these two papers they describe the same space only naming it differently. They also note their sinus may not be present in 25% of cases because of high-placed jugular bulbs.

Then again, the history of the minute recesses of retro- and hypotympanum is far more complicated. Although we have to acknowledge Bruce Proctor for the first attempts of naming it, one must include his antecessors. Interestingly, the story starts in Modena, Italy, with young Antonio Scarpa in 1772 [24]. His magnificent work was translated in 1962 by Sellers and Anson as a contribution to the work of Theodore Bast in the 3rd year after he had passed. Thanks to them, we know Scarpa’s description: “And as for the round window, the foramen indicated by the letter “D” [here he referred to Tables of Eustachius] appears to represent a peculiar sinuosity in front of the window rather than the window itself” [25].

We also found Arthur Cheatle describing the spaces of retrotympanum in 1907. “The fossa on each side of the pyramid varies greatly”, he wrote, ”(…) the sinus tympani is often comparatively great extent running backwards (…). It may dip down forming a pocket and having sulcus jugularis as an inner wall below the vestibule.” [11].

Then Theodore Bast, a brilliant professor of anatomy, complained in 1952, “In recent literature and textbooks, the region of cochlear fenestra has not been critically considered”. He continues with the description: “The fossula of the cochlear window (fossula fenestrae cochlea, fossula fenestrae triquetrae, canaliculus fenestrae rotunda or niche of the cochlear window) is an irregularly prismatic recess, with rounded angles, which extends from a point near the bottom of the middle ear upward and inward beneath the arch of first turn of the cochlea” [5].

Dworacek in his report on microscopic anatomy of the tympanic cavity from 1960 stated “The round window or its niche is limited from above and below by two branches of the promontorium which in various variants gently slope backwards or downwards (…). The variety of shape is particularly great here. Depending on whether the frame is smooth or jagged, the access to the window niche also has an irregular shape” [12].

When Omulecki wrote about round window vicinity in 1963, he described the internal and external openings of round window niches. The internal opening was closed by a secondary tympanic membrane, and the external led to an unnamed recess he characterized as follows: “When looking straight at the medial wall of the tympanic cavity, there is a distinct depression between the posterior wall of the tympanic cavity and the niche of the round window” [19].

Finally, a few years before Proctor, in 1982, Garcia et al. [13] investigated styloid prominence and its variations. In their opinion, styloid prominence is the structure of importance limiting the posterior recesses of the tympanic cavity. However, they consequently used the term “fossula of cochlear fenestra” throughout the paper as the name of the recess located medially to styloid prominence [nowadays styloid eminence], anteroinferior to sinus tympani and close to the round window. They also reported the area is difficult to access because of “innumerable irregular bony trabeculae” joining the styloid complex and promontory.

All these descriptions fit the space we have been investigating.

## Methodology discussion

Apart from the complicated history of the definition of the subtympanic sinus, the borders, shape, and position are now well established. In our work, we have decided to use computed tomography scans because of their availability in great numbers, unlike anatomical specimens. The number of samples was the problem of previously conducted research, as shown in Table 3. Our work is the first one using percentage numbers as they should be used, i.e., in a group of more than 100 subjects. Geneci and Ocak point out that STS needs to receive more attention regarding its clinical significance [14]. The qualitative and quantitative studies on area concamerata (future STS) were barely conducted before 2010. The main problem was visualizing the recess and consensus about its borders. Only Savic and Djeric report the inferior hypotympanic sinus present in 26.5% of their 50 examined temporal bones and its depth as about 2 mm. The measurement accuracy was 0.5 mm. Little was known regarding anatomical variations of bony projections between styloid complex, pyramidal eminence, and round window area [23].

**Table 3:** Comparison of the percentage distribution of individual types of sinuses in the literature and the analyzed material

STS type	Anschuetz et al. [3]	Bonali et al. [9]	Hool et al. [15]	Geneci and Ocak [14]	Our material		
Method	HRCT and direct examination	HRCT and direct examination	HRCT and intraoperative examination	microCT and direct examination	HRCT		
	Non-diseased temporal bones	Non-diseased temporal bones	diseased temporal bones	Non-diseased temporal bones	Non-diseased temporal bones		
Age group	Adults	Adults	Adults	Adults	Children Age 4–60 months	Children Age 4–24 months	Children Age 25–60 months
Number of samples	*n* = 29	*n* = 30	*n* = 59	*n* = 40	*n* = 280	*n* = 196	*n* = 84
A	72%	47%	69%	72.5%	59.3%	53.6%	72.6%
B	21%	47%	24%	20%	30.7%	34.7%	21.2%
C	7%	6%	7%	7.5%	10%	11.7%	6%

After the introduction of the term subtympanic sinus, there were only three papers investigating it in terms of a visualization in HRCT [3, 9, 15] and one using microCT [14]. Anschuetz et al. [3] introduced the classification of the STS in CT scans similarly to the classification of tympanic sinus introduced by Marchioni et al. [17] and facial sinus by Alicandri-Ciufelli et al. [1]. In their work, they described the relationship of the STS with the mastoid part of the facial nerve, and in order to avoid mistakes, they did three-dimensional reconstructions of scanned bones. The images they provided are clear and suggest they assessed the STS type on the level of the round window niche when the anterior pillar may be found, although it is not directly written in the methodology section. In our study, we describe the detailed method of interpretation of the STS area. We also suggest checking five scans superiorly and five scans inferiorly in order to avoid mistakes and taking measurements of sinus tympani. Especially that Bonali et al. [9] found the absence of subiculum in up to 14% of cases in which the tympanic sinus and subtympanic sinus merge into one large recess.

Anschuetz et al. [3] and Bonali et al. [9] correlated endoscopic findings on cadavers with CT scans. They used only specimens with no history of ear diseases. The first team brought attention to pneumatisation and the position of the jugular bulb. It is consistent with observations of Proctor [22] and those before him which suggest correlation of retrotympanic sinuses depth with overall pneumatization of the mastoid or whole temporal bone [5] and also the pneumatization of the sinuses between each other [11]. Also, a high-riding jugular bulb may narrow the entrance to the subtympanic sinus or alter its shape [11, 16, 23]. That is why we decided to check the air cell tracts medially and posteriorly to the STS. The 5 mm radius idea of solid bone to state there is no pneumatisation was adopted from a recent study on the jugular bulb and its vicinity by Tudose et al. [28].

Bonali et al. [9] suggest that underdeveloped pneumatization in disease-affected ears may alter the configuration of type and depth of the tympanic sinus, facial sinus, and subtympanic sinus. Hool et al. [15] acknowledge the difference in the anatomical variability of diseased and healthy ears and set up research on preoperative HRCT and endoscopic findings in patients undergoing endoscopic ear surgery. They also state that even neuroradiologists may find it challenging to analyze retrotympanic cavities. Researchers do not provide the kappa coefficient and instead propose to meet, seek for resolution, and find consensus when discrepancies are detected. In our study, we used the same idea of consensus meetings and measurements accepted by both researchers involved in the analysis.

Geneci and Ocak [14] have rightly pointed out the small number of samples in previous studies. In our study, we have addressed this issue by providing a sufficient number of samples for a proper statistical analysis. We chose to investigate the STS type and measurements during the pneumatisation process in normal conditions, specifically using CT scans of healthy children. This focus on normal conditions is crucial as it can provide valuable insights into the clinical implications of subtympanic sinus anatomy.

## Discussion of the results

Marchioni et al. [16] stated that the subtympanic sinus might be, as often as sinus tympani, the place of residual cholesteatoma. Through years and consecutive papers, he classified almost all structures vital for endoscopic ear surgery. Marchioni also emphasized that sinus depth and its relations to the facial nerve are essential factors when planning the removal of inflammatory tissues. When a surgeon intends to perform an endoscopic cholesteatoma excision, one should gather as much information as possible. Computed tomography is the perfect tool to collect such data, and we support the opinion that it is obligatory to interpret CT scans before cholesteatoma surgery. In children, the preoperative assessment is also important before cochlear implantation.

Endoscopic visualization of the fundus of the type C tympanic sinus, facial sinus, and subtympanic sinus is very difficult [9]. The deeper the medial sinuses are, the greater the risk of injuring the facial nerve [11, 17]. Until now, it was proven the type C configuration of the tympanic sinus [17, 31], facial sinus [1, 32] and subtympanic [3, 14] is the least common in healthy adults. Also, in healthy children, type C tympanic sinus [30] and facial sinus [32] are the least common. Our work is the first to investigate subtympanic sinus regarding its types in such numbers and children. Our findings are a bit different from the distribution reported by other researchers (Table 3.), probably due to the sample size. As the overall distribution is less favorable for type A (shallow) STS, it is interesting how the percentage of type A STS grows in a group of older children (from 59 to 72%). The distribution of particular types in group of older children is quite similar as in the groups of healthy ears investigated by Anschuetz et al. [3] and Geneci and Ocak [14]. We believe it may be due to the development of pneumatization routes around the facial nerve and the relative position change of the mastoid part of the facial nerve and the otic capsule. The interesting fact is the distribution of types in diseased ears in the group analyzed by Hool et al. [15] is similar to our group of children and adult groups [3, 14]. There is a need for research on healthy and diseased adults on a larger sample size to confirm these findings.

Anschuetz et al. [3] proposed their new classification based on radiological classifications for the tympanic sinus [17] and facial sinus [1]. Geneci and Ocak [14] put in doubt the necessity of having the classification for the STS. They state that most STS do not exceed the level of facial nerve and therefore encourage one to put an effort into further studies. On the other hand, we found statistically significant differences in depth between types of subtympanic sinuses. These results support the qualitative categorization of STS into types A, B, and C.

Only one paper provides the depth measurements written by Geneci and Ocak [14], who analyzed micro CT scans. They described the depth measurement as distance from the STS’s medial border and the FN’s mastoid part. The exact points chosen for measurements are not defined, so the comparison is limited. The means and ranges are lower in their group (Table 4.), and it may be because they used temporal bones of adults after completion of the pneumatization process in healthy conditions and stable spatial relationship between the facial nerve, otic capsule, and air cell tracts around.

**Table 4:** Comparison of the STS mean depth and min-max values in individual types of sinuses between findings of Geneci and Ocak [14], and our material; in [mm]

STS type	Geneci and Ocak [14]	Our whole material	Children age 4–24 months	Children age 25–60 months
A	2.35 (1.43–6.43)	2.69 (1.10–4.79)	2.67 (1.23–4.18)	2.72 (1.10–4.79)
B	2.12 (1.54–2.86)	3.76 (1.95–6.27)	3.79 (1.95–6.27)	3.65 (2.22–5.05)
C	3.34 (3.25–3.37)	5.06 (3.31–8.43)	4.88 (3.31–8.43)	5.92 (4.69–6.99)
Total	2.38 (1.43–6.43)	3.26 (1.10–8.43)	3.32 (1.23–8.43)	3.11 (1.10–6.99)

As there is no information in the literature on the topic of STS width, we cannot compare it with other research. It seems the STS has a broader entrance (width) than sinus tympani in children [20, 30] and adults [31]. Facial sinus entrance is generally wider [7, 32]. However, its access is not so difficult because its lateral position about the facial nerve and scutum may always be removed. The shallow STS is more accessible than the sinus tympani as it is anterior and has a wider entrance, but both ST and STS type C may be inaccessible endoscopically, no matter the width of the entrance. That is why the aural surgeon must be able to convert from an endoscopic to a microscopic approach. A microscopic alternative for STS is a retrofacial approach via the transmastoid route, as with deep tympanic sinuses [3].

Hool et al. [15] state that in the case of hypopneumatization of the mastoid, the recesses of the retrotympanum may be shallow (type A). On the other hand, Baklaci et al. [4] found that well-pneumatized mastoid in adults was highly related with a deep and posteriorly positioned ST with respect to FN. As there is no consensus on the classification system of pneumatisation in children, we used Allam’s [2] universal proposition. We found the presence of both retrofacial and hypotympanic air cell tracts increased in the group of older children. That proves the ongoing development of pneumatisation with age. However, no statistically significant correlation was demonstrated between the depth and the presence of these air cell tracts. Hool et al. [15] also compared available data on recesses of retrotympanum by adding all sinuses according to type, i.e., type A ST, FS, and STS, and so on, and they presented percentage values in relation to the summed population from four papers. As we know from extensive group studies on adults and children, the particular sinuses are not characterized by normal distribution. Even though we analyze the same factors as depth and relationship to the mastoid facial nerve, the percentage distributions vary. We recommend assessing each sinus separately and not predicting the type of one sinus based on the other. Lack of information in these regards leads to the idea of proposing radiomorphologic profiles using depth, width, and type of particular sinuses as proposed in the model of nonsyndromic sagittal craniosynostosis assessment [26]. There may be a pattern of particular coexistence of retrotympanic recesses. In further studies, we would like to check the configuration and coexistence of the tympanic sinus, facial sinus, subtympanic sinus, and subcochlear canaliculus at the same time. Four significant recesses are essential for surgical planning, and each has three possible configurations, so we assume a square matrix 4 × 4. In each matrix window, there are 3 options. We want to check whether there are any dominant patterns, including all recesses simultaneously in the same subject.

The inferior wall of the tympanic cavity is also called the jugular wall because of its close relationship to the jugular bulb. Many researchers appreciated the role of the jugular bulb in establishing the final shape of the transitional area between retro- and hypotympanum [8, 11–13, 16]. There are quite a few distinct classifications of high-riding jugular bulbs, but one of the possible variants is JB protruding to the tympanic cavity. It may occur, mainly when the domination of JB is observed. Savic and Djeric [23] found the dome of the JB protruding into the hypotympanum in about 25% of examined bones. The cavity of the hypotympanum is significantly reduced or even missing in such a situation. In our group, during ongoing pneumatization, we have found JB modeling the STS in up to 22% of older children with no side correlation. It is similar to the results of Savic and Djeric in terms of the general interpretation of the role of JB in this area. Further investigations are needed to check whether there is a side correlation after the JB establishes its final shape in adults.

The radiologic anatomy of the retrotympanum is very difficult to analyze. Burd et al. [10] highlighted that there is a need for close cooperation between otologists and radiologists. We agree that with this remark, there should be consensus meetings. We think radiologists would benefit from operating room visits, operative video explanations, and participation in cadaver labs. On the other hand, otologists would appreciate more hours with image interpretation and three-dimensional reconstructions. One may be advised because the current study from Beckmann et al. [6] suggests an overestimation of cholesteatoma in CT scans compared to intraoperative findings. It is yet another cause for the thorough and long-lasting study of microscopic, endoscopic, and radiological anatomy.

### Further investigations

Our next goal is to correlate these findings with specimens or intraoperative endoscopic ear surgery videos to cover healthy and diseased temporal bones. Further studies in CT scans in adults would also be desired to check the distribution of particular STS types after the pneumatisation development is completed. In contrast to the clinical CT examination it would also be beneficial to consider research that examine the morphology of the STS using micro-CT, as this technique offers a huge potential for detailed 2D and 3D imaging of the small compartments in the tympanic cavity [27]. However it is limited to the examination of dry bone samples.

## Limitations of the study

In our opinion, the main limitation of our study is the lack of clinical information regarding the subjects’ medical history. We could only exclude scans with visible pathologies. We also did not have an opportunity to compare the results between children and adults as we simply do not have access to computed tomography scans of temporal bone nowadays. The inter and intraobserver were not performed as the CT scans were consensus assessed and agreed upon by two anatomists experienced in temporal bone radiology. Although we have undertaken measures to describe the STS with the most accuracy (see methodology discussion), it is still research done purely on CT scans and it is per se subjective. While classification of the STS in multiplanar reconstruction models remains subjective, it may contribute to a better spatial orientation in the middle ear anatomy, thereby, have important implications during planning of middle ear endoscopic surgery. The number of samples, the age range, and the variation of ethnic background are limited in this study, limiting the results’ validity to cohorts similar to the cohort examined in this study. Future classical in vivo anatomical work would be required – either on temporal bone specimens in more significant numbers than in the past or by assessment of intraoperative videos.

## Conclusions

1. We have proven that the qualitative classification of the subtympanic sinus into types A, B, and C introduced by Anschuetz et al. [3] is justified by statistically significant differences of depth between individual types of subtympanic sinuses in our group.
2. The type A subtympanic sinus is dominant in the whole pediatric group; however, the percentage increased significantly in the group of older children. The percentage of type B and C STSs decreased with age.
3. The width of the STS varies between genders but not between age groups and sides. Specific ranges of the STS depth are correlated with types. However, due to overlapping ranges, it is not possible to define the STS type by knowing only the depth value.
4. Although the pneumatisation of retrotympanic and hypotympanic cells increases in the group until 5 years of age, the correlation between depth or the generally final shape of the subtympanic sinus is not clear yet.
5. The jugular bulb position may change the final shape of the STS as it is closely related to the retro- and hypotympanum.
6. The radiological assessment of subtympanic sinus may be clinically important in minimally invasive ear surgery.
7. The area currently known as sinus subtympanicus was also described as sinus hypotympanicus or area/sinus concamerata. Earlier, the researchers did not distinguish between a round window niche or fossula for the round window – the area was described in detail and divided into subareas because of advancements in endoscopic ear surgery and better imaging.

## Data Availability

No datasets were generated or analysed during the current study.
